# Synergistic Cr(VI) Reduction and Chloramphenicol Degradation by the Visible-Light-Induced Photocatalysis of CuInS_2_: Performance and Reaction Mechanism

**DOI:** 10.3389/fchem.2022.964008

**Published:** 2022-07-12

**Authors:** Chaosheng Zhu, Jingyu Li, Yukun Chai, Yongcai Zhang, Yunlin Li, Xiangli Zhang, Jin Liu, Yan Li

**Affiliations:** ^1^ Zhoukou Key Laboratory of Environmental Pollution Prevention and Remediation, School of Chemistry and Chemical Engineering, Zhoukou Normal University, Zhoukou, China; ^2^ School of Chemistry and Chemical Engineering, Yangzhou University, Yangzhou, China; ^3^ College of Chinese Language and Literature, Zhoukou Normal University, Zhoukou, China; ^4^ Henan Key Laboratory of Rare Earth Functional Materials, International Joint Research Laboratory for Biomedical Nanomaterials of Henan, Zhoukou Normal University, Zhoukou, China

**Keywords:** CuInS_2_, Cr(VI) reduction, chloramphenicol degradation, synergistic effect, visible light photocatalysis

## Abstract

Despite significant scientific efforts in the field of water treatment, pollution of drinking water by toxic metal ions and synthetic organic compounds is becoming an increasing problem. The photocatalytic capabilities of CuInS_2_ nanoparticles were examined in this study for both the degradation of chloramphenicol (CAP) and the reduction of Cr(VI). CuInS_2_ nanoparticles were produced using a straightforward solvothermal approach and subsequently characterized by many analysis techniques. Simultaneous photocatalytic Cr(VI) reduction and CAP oxidation by the CuInS_2_ nanoparticles under visible-light demonstrated that lower pH and sufficient dissolved oxygen favored both Cr(VI) reduction and CAP oxidation. On the basis of active species quenching experiments, the possible photocatalytic mechanisms for Cr(VI) conversion with synchronous CAP degradation were proposed. Additionally, the CuInS_2_ retains a high rate of mixed pollutant removal after five runs. This work shows that organic contaminants and heavy metal ions can be treated concurrently by the visible-light-induced photocatalysis of CuInS_2_.

## Introduction

Industries such as electroplating, mining, leather tanning and electronics manufacturing use large amounts of chromium compounds, leading to serious water pollution (Djellabi et al., 2019; Ge et al., 2021; Taha et al., 2021). Due to its carcinogenic, teratogenic, and transportable properties, Cr(VI) poses a substantial hazard to both the environment and human health (Wei et al., 2017; Djellabi et al., 2021; Xiong et al., 2022). Furthermore, antibiotics are used widely to treat bacterial illnesses, resulting in widespread contamination of aquatic ecosystems, including surface water and groundwater (Abdurahman et al., 2021; Bouyarmane et al., 2021; Yang et al., 2022). Because the biological toxicity of such compounds endangers the aquatic organisms and human health, a growing emphasis is being placed on their efficient removal (Qiu et al., 2022). Chloramphenicol (CAP) is a broad-spectrum antibiotic that can be used to treat a wide range of bacteria and viruses (Sun et al., 2022). Ingestion of CAP-contaminated water may lead to the growth of antibiotic-resistant bacteria and a reduction in medullary hematopoiesis function (Yu et al., 2019). Given the inadequacy of conventional sewage treatment plants in eliminating CAP, the total removal of these antibiotic compounds from water is a major concern. In reality, heavy metals and organic contaminants coexist in the same environment quite regularly.

A variety of strategies for removing CAP and Cr(VI) have already been documented to date, including adsorption, advanced oxidation processes, and biological treatment. Because of its low cost, safety, and great efficiency, photocatalysis-based advanced oxidation technique has gotten a lot of interest in the field of organic wastewater purification (Liu et al., 2009; Han et al., 2018; Yang et al., 2021a). Several photocatalysts have also been explored for the degradation of CAP, such as jarosite, LSCO_5_, and SmVO_4_/g-C_3_N_4_ composite (Wang et al., 2021b; Leeladevi et al., 2021; Wu et al., 2022). Moreover, controlling Cr(VI) pollution through photocatalytic reduction is a viable option. A number of photocatalysts such as Fe_2_O_3_, Bi_2_MoO_6_, g-C_3_N_4_, SnS_2_ and their composites have been shown to be capable of reducing Cr(VI) to less harmful Cr(III) under visible-light irradiation (Zhang et al., 2016; Wu et al., 2020; Liu et al., 2022b; Ge et al., 2022; Tao et al., 2022). The mechanism indicates that the conduction band and valence band of the photocatalyst play the roles of reduction and oxidation, respectively, which provides the possibility of simultaneous removal of Cr(VI) and CAP by reduction and oxidation reactions in the same photocatalytic system. Thus, the development of effective and efficient photocatalysts is required for synergistic photocatalytic reduction of Cr(VI) and degradation of CAP.

Owing to its durability, low toxicity, appropriate band gap, and good solar energy conversion efficiency, I-III-VI ternary metal sulfide semiconductors have received a lot of research attention thus far (Li et al., 2018; Zhang et al., 2021). Copper indium sulfide (CuInS_2_) is a promising I-III-VI_2_ ternary chalcopyrite material with a wide range of advantages for photocatalytic and photovoltaic applications (Yang et al., 2021b; Spera et al., 2022; Wang et al., 2022). Its conduction band is made up of In 5s orbitals, and its valence band is made up of S 3p orbitals, resulting in a small band-gap (1.53 eV for the bulk CuInS_2_) (Chumha et al., 2020; Guo et al., 2021). It has been investigated as a photocatalyst for CO_2_ reduction (Xu et al., 2018), organic pollutant degradation (Guo et al., 2019; Kaowphong et al., 2019), and nitrate ion reduction (Yue et al., 2016), etc. However, it has not been applied to the field of synergistic photocatalytic elimination of Cr(VI) and organic pollutants.

Here, CuInS_2_ nanoparticles were prepared using a one-step hydrothermal technique, and investigated as a photocatalyst in the concurrent elimination of Cr(VI) and CAP under visible-light irradiation. Furthermore, on the grounds of different characterizations and theoretical analysis, the synergistic removal effect of pollutants and photocatalytic reaction mechanism in the Cr(VI)-CAP coexistence system were explored and discussed in detail.

## Experimental

### Materials

All the reagents (analytical grade) were purchased and used as received from Sinopharm Chemical Reagent Co., Ltd. Throughout the study, all solutions were prepared with ultrapure water (18.2 MΩ⋅cm).

### Synthesis of CuInS_2_

4 mmol CH_3_CSNH_2_, 2.0 mmol In(NO_3_)_3_·H_2_O, and 2.0 mmol Cu(NO_3_)_3_·3H_2_O were dissolved in 40 ml anhydrous ethanol, and stirred with a magnetic stirrer for 60 min to generate a homogenous dark brown suspension. The solution was then transferred into a 100 ml Teflon-lined stainless steel autoclave and heated at 180°C for 2 h. After cooling to ambient temperature naturally, the precipitate was centrifuged, washed alternately with ethanol and water, and dried at 80°C for 24 h.

### Characterization of the Synthesized CuInS_2_

An X-ray diffractometer (XRD, PANalytical B.V.) was used to determine the phase of the as-obtained product. Field emission scanning electron microscope (FE-SEM, Japan Hitachi LTD. S4800) and transmission electron microscopy (TEM, Japanese electronics JEM-2100PLU) were used to characterize the products’ microstructure. The point of zero charge (pH_pzc_) was measured by pH drift method. X–ray photo-electron spectroscopy (XPS) was performed on a PHI 5000C ESCA System. Using a UV–Vis spectrometer (UV-2450, Shimadzu, Japan) equipped with an integrating sphere attachment and BaSO_4_ as a reflectance standard, UV–Vis diffuse reflectance spectrum of the dry-pressed disk sample was acquired.

### Photocatalytic Tests

The photocatalytic performance of the synthesized CuInS_2_ was determined by monitoring the simultaneous photocatalytic reduction of Cr(VI) and degradation of CAP in aqueous solution under visible-light illumination. The photocatalytic experimental setup (PL-02, Beijing Precise Technology Co., Ltd.) contains a Xe arc lamp (1000 W) with a 400 nm cutoff filter, a set of cylindrical quartz reactors (80 ml), and a cold trap to keep the temperature of reaction solution constant. By dissolving K_2_Cr_2_O_7_ and CAP in ultrapure water or diluting the stock solution with ultrapure water, varied concentrations of Cr(VI) and CAP solutions were obtained. The pH of the solution was adjusted to the anticipant value using a concentrated solution of NaOH or H_2_SO_4_. A set of tests were performed to investigate the photocatalytic Cr(VI) reduction and CAP degradation over CuInS_2_ at various solution pHs and Cr(VI)/CAP ratios. Cr(VI) concentration was determined by the modified N, N-diphenylcarbazide spectrophotometry method (Li et al., 2021). The concentration of CAP in the solution was determined using high-resolution liquid chromatography (HPLC, Accela, Thermo Scientific, United States) equipped with an XB-C18 column (4.6250 mm, 5 m, Yuexu, China) and a UV detector. The mobile phase consisted of a 50: 50 mixture of acid aqueous solution (0.1% acetic acid) and acetonitrile. The chromatograms of CAP were achieved using a flow rate of 1.0 ml min^−1^, an injection volume of 10 μl, and 277 nm as UV detection wavelength. The column was maintained at a temperature of 30°C. For each time measurement, approximately 4 ml of the aqueous solution was withdrawn from the cylindrical quartz reactors and filtered through a 0.45 nm filter to get rid of catalysts.

### The Photocatalytic Reaction Kinetics Model

The photocatalytic Cr(VI) reduction and CAP degradation over CuInS_2_ under a variety of operating conditions were studied by pseudo-first-order kinetics, which was expressed as Eq. 1 (Mao et al., 2022):

$$\mathrm{In}\left(\frac{C_{0}}{C}\right)=\mathrm{Kt}$$ (1)

Here, C_0_ represents the concentration of Cr(VI) or CAP following the adsorption-desorption equilibrium, C represents the concentration of Cr(VI) or CAP at the irradiation time of t min, t represents the irradiation time (min), and k represents the apparent rate constant (min^−1^).

## Results and Discussion

### Characterization of the As-synthesized CuInS_2_

**FIGURE 1:**
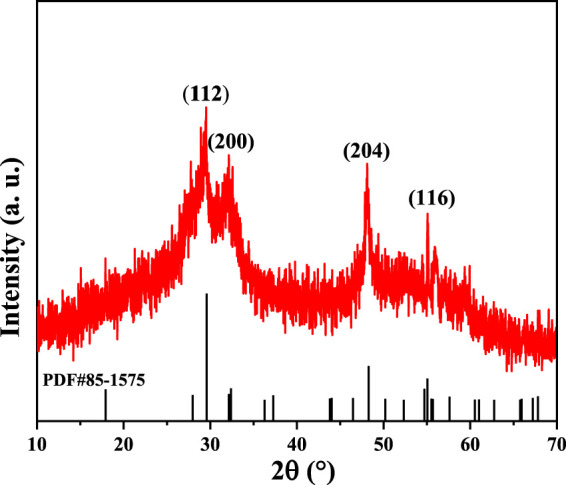
XRD pattern of our synthesized CuInS_2_.

XRD was used to determine the crystalline structure of our CuInS_2_ product. As illustrated in Figure 1, there appear the diffraction peaks at 2θ = 28.1°, 32.2°, 47.1°, and 55.1°, which are respectively indexed to the (112), (200), (204), and (116) crystal planes of chalcopyrite structure CuInS_2_ (JCPDS card No. 85–1575). The weak and wide diffraction peaks in the XRD pattern suggests that our CuInS_2_ product has poor crystallization.

**FIGURE 2:**
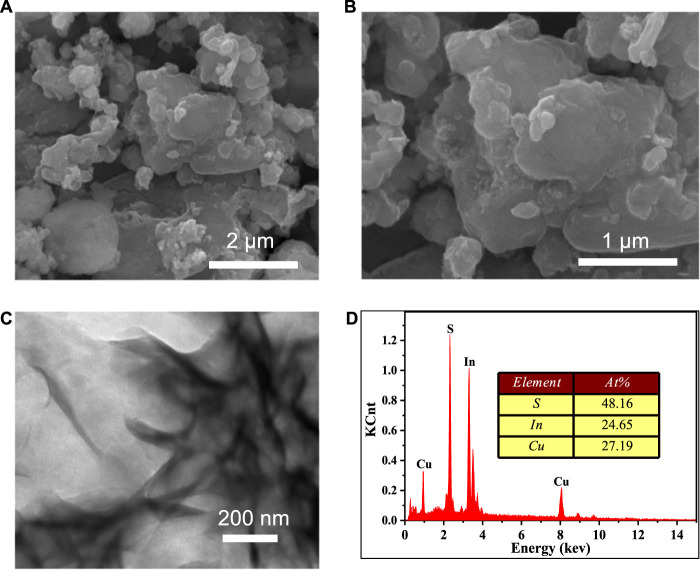
**(A,B)** SEM images, **(C)** TEM image and **(D)** EDS spectrum of our synthesized CuInS_2_.

SEM and TEM were used to investigate the morphology and size of our produced CuInS_2_. As illustrated in Figures 2A–C, the CuInS_2_ sample exhibits a sheet stacking structure with dimensions ranging from 200 to 5,000 nm. The flake structure of CuInS_2_ allows for the exposure of more active sites, which is highly advantageous for photocatalytic reactions. The EDS spectrum (Figure 2D) demonstrated that the prepared sample comprised the Cu, In, and S elements with a Cu, In, and S atomic ratio of around 1: 1: 2, confirming the formation of CuInS_2_.

**FIGURE 3:**
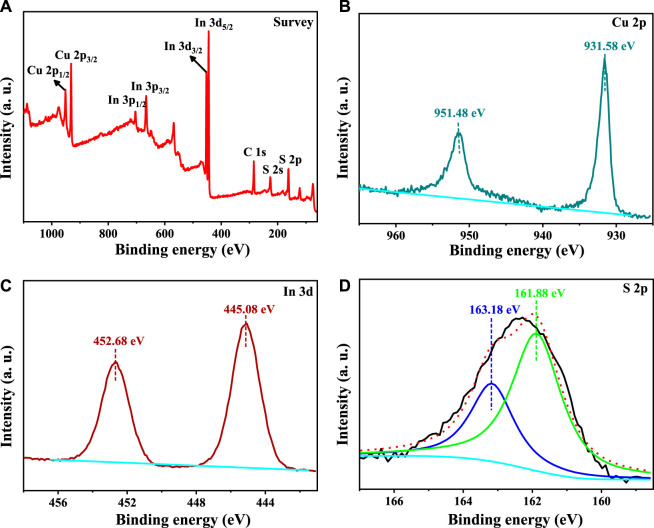
**(A)** Survey, **(B)** Cu 2p, **(C)** In 3days, and **(D)** S 2p XPS spectra of our synthesized CuInS_2_.

The whole XPS spectrum of our synthesized CuInS_2_ is shown in Figure 3A, which reveals the existence of Cu, In, S 2p, and adventitious C in this sample. The Cu 2p XPS spectrum of our synthesized CuInS_2_ is displayed in Figure 3B, which shows sharp peaks with binding energies of 951.48 eV (Cu 2p_1/2_) and 931.58 eV (Cu 2p_3/2_), respectively. There is no characteristic Cu^2+^ peak at 934.3 eV, indicating that only the Cu^+^ oxidation state is present in our synthesized CuInS_2_ (Li et al., 2016; Yue et al., 2017). The In 3 days XPS spectrum in Figure 3C shows two peaks at 452.68 eV and 445.08 eV, which match with In 3d_3/2_ and In 3d_5/2_, respectively. These binding energy values indicate that In is in the In^3+^ oxidation state (Yang et al., 2015). The S 2p XPS spectrum (Figure 3D) was fitted into two peaks at 163.18 eV (S 2p_1/2_) and 161.88 eV (S 2p_3/2_), which are attributed to S^2-^ bonded to In or Cu in CuInS_2_ (Liu et al., 2019; Guo et al., 2021).

**FIGURE 4:**
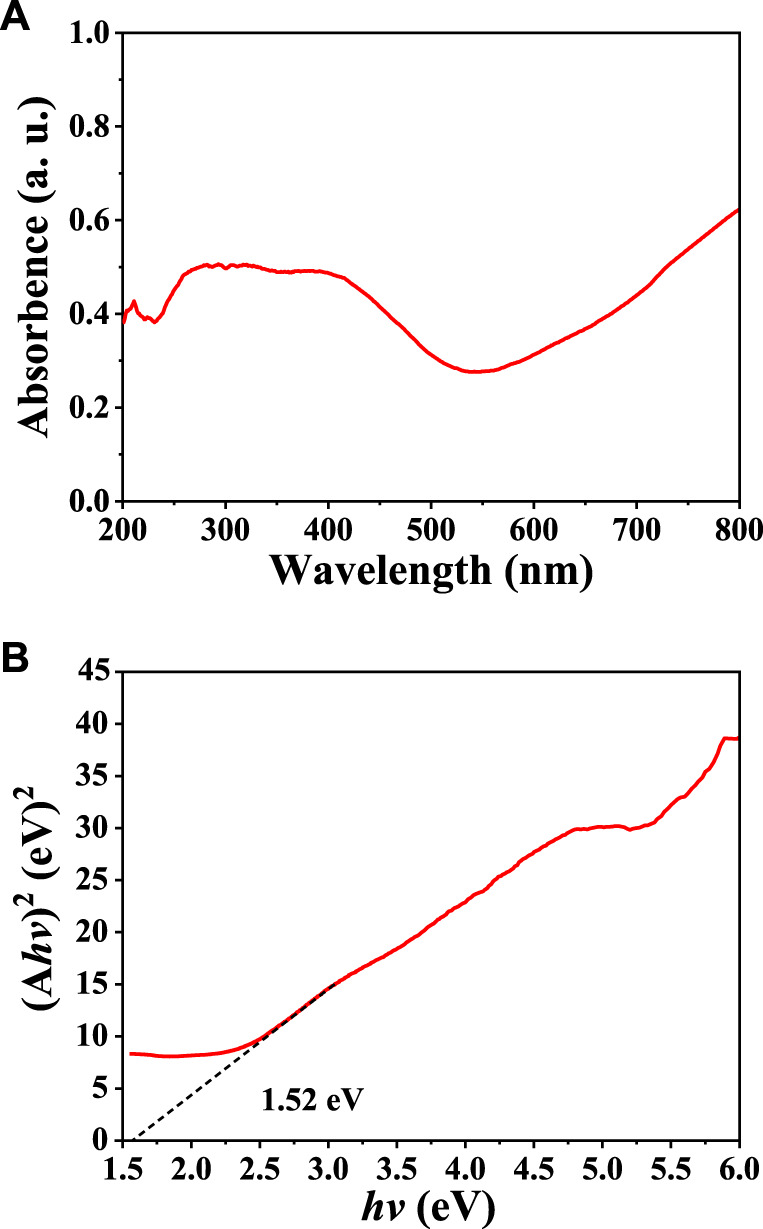
**(A)** UV–Vis diffuse reflectance spectrum and **(B)** Tauc plot for obtaining the E_g_ value of our prepared CuInS_2_ sample.

The UV-Vis diffuse reflectance spectrum of our synthesized CuInS_2_ was used to analyze its optical absorption property and band gap energy (E_g_). As illustrated in Figure 4A, the CuInS_2_ product demonstrates distinct absorption of visible light between 550 and 800 nm. The following formula (Eq. 2) can be used to estimate the E_g_ of the CuInS_2_ product (Liu et al., 2022a):

$${\left(\alpha hv\right)}^{n}=K\left(\mathrm{hv-}\mathrm{Eg}\right)$$ (2)

where *h* is the Planck’s constant, α is the absorption coefficient, k is the constant, *v* is the light frequency, *n* = 1/2 for an indirect band gap semiconductor, or *n* = 2 for a direct band gap semiconductor. Since absorbance (A) is directly proportional to the absorbance coefficient (α), the same E_g_ value can be obtained by replacing α with A. CuInS_2_ is a direct band gap semiconductor. As shown in Figure 4B, by projecting the linear part of its (A*hv*)^2^ vs. (*hv*) plot to zero, the E_g_ value of CuInS_2_ is obtained to be 1.52 eV, which is close to the reported values in the literature (Guo et al., 2019; Chumha et al., 2020). This means that the CuInS_2_ nanomaterial could be used as a visible-light-driven photocatalyst.

### Effect of Solution pH on Cr(VI) Reduction and CAP Degradation

**FIGURE 5:**
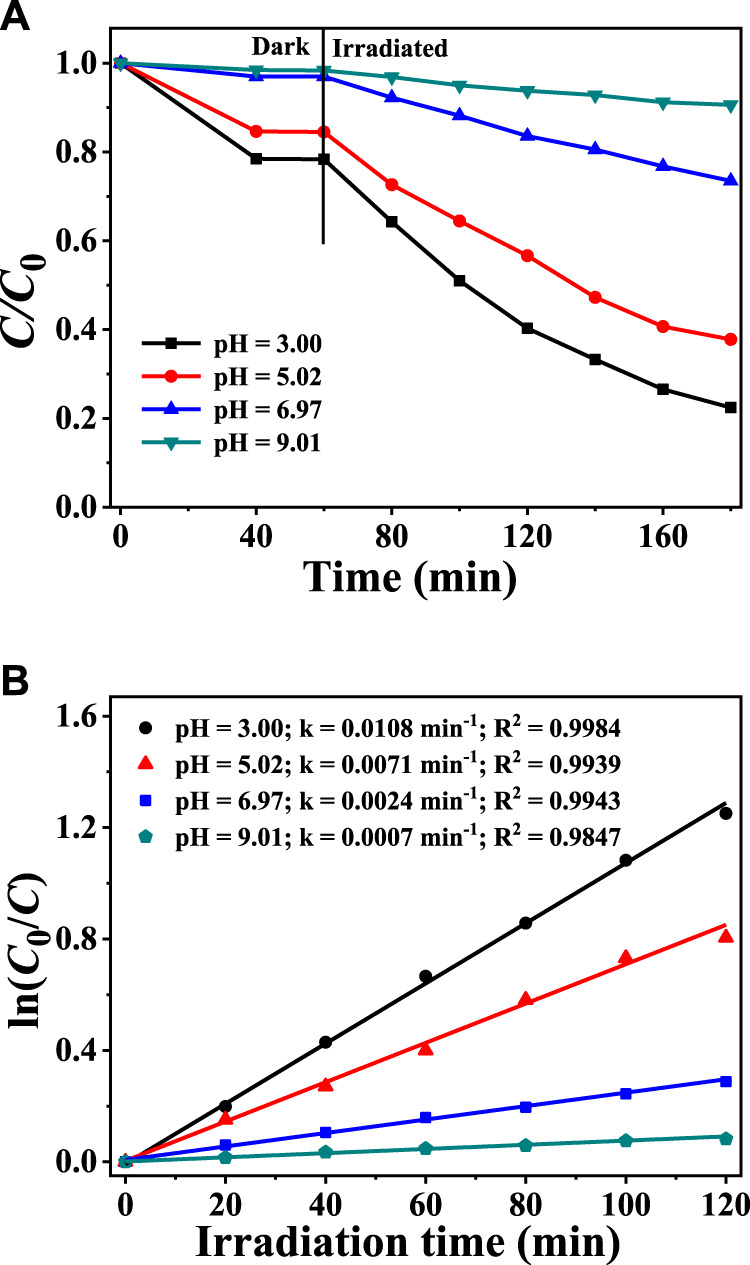
**(A)** Adsorption and photocatalytic reduction of Cr(VI) over CuInS_2_ in the mixed solution of Cr(VI) and CAP at different pH conditions **(B)** Corresponding kinetics plots for the photocatalytic Cr(VI) reduction reactions in **(A)**. ([Cr(VI)] = 10 mg/L, [CAP] = 10 mg/L, [catalyst] = 0.2 g/L).

Since the solution pH can modify both the acid-base environment and the existing forms of Cr(VI), it is well established that the solution pH values exert a significant effect on photocatalytic activities (Zhang et al., 2018; Mangiri et al., 2021; Kumar et al., 2022). The effects of varying the initial pH on Cr(VI) reduction are demonstrated in Figures 5A,B According to Figure 5A, the adsorption equilibrium between catalysts and Cr(VI) can be attained after they were mixed for 40 min, because the Cr(VI) concentration did not decrease when the adsorption duration was further raised to 60 min. When the pH value decreased from 7–9 to 5–3, the Cr(VI) adsorption capacity of CuInS_2_ increased rapidly. The pH_PZC_ of prepared CuInS_2_ is determined to be 5.68, so in the solutions with pH less than 5.68, the surface charges of CuInS_2_ are positive, which is conducive to the adsorption of Cr_2_O_7_^2−^, HCrO_4_^−^ and CrO_4_^2−^ (Xu et al., 2020). When the photoreduction process began, the reduction rates of Cr(VI) significantly increased with the lengthening of the irradiation period. In addition, the decrease of the solution pH from 9.01 to 3.00 also resulted in the faster reduction rates of Cr(VI). According to Figure 5B, when the solution pH value is 3.0, the *k* value (0.0108 min^−1^) of the photocatalytic Cr(VI) reduction over CuInS_2_ is the largest, which is about 15.43 times as that (0.0007 min^−1^) at pH 9.01. One reason for this is that a lower pH promotes Cr(VI) adsorption on the photocatalyst, hence accelerating photocatalytic conversion. Another possibility is that lowering the pH value of reaction solution raises the chromate reduction potential. For example, one pH unit lower results in a rise in the standard reduction potential by 0.138 V (Zhang et al., 2017). So the E(Cr(VI)/Cr(III)) value increases from 0.24 to 1.06 V as the pH of the solution decreases from 9.00 to 3.00. From the viewpoint of both kinetics and thermodynamics, the photocatalytic Cr(VI) reduction rate would be enhanced in the lower pH solution (Marinho et al., 2016; Zhang et al., 2022).

**FIGURE 6:**
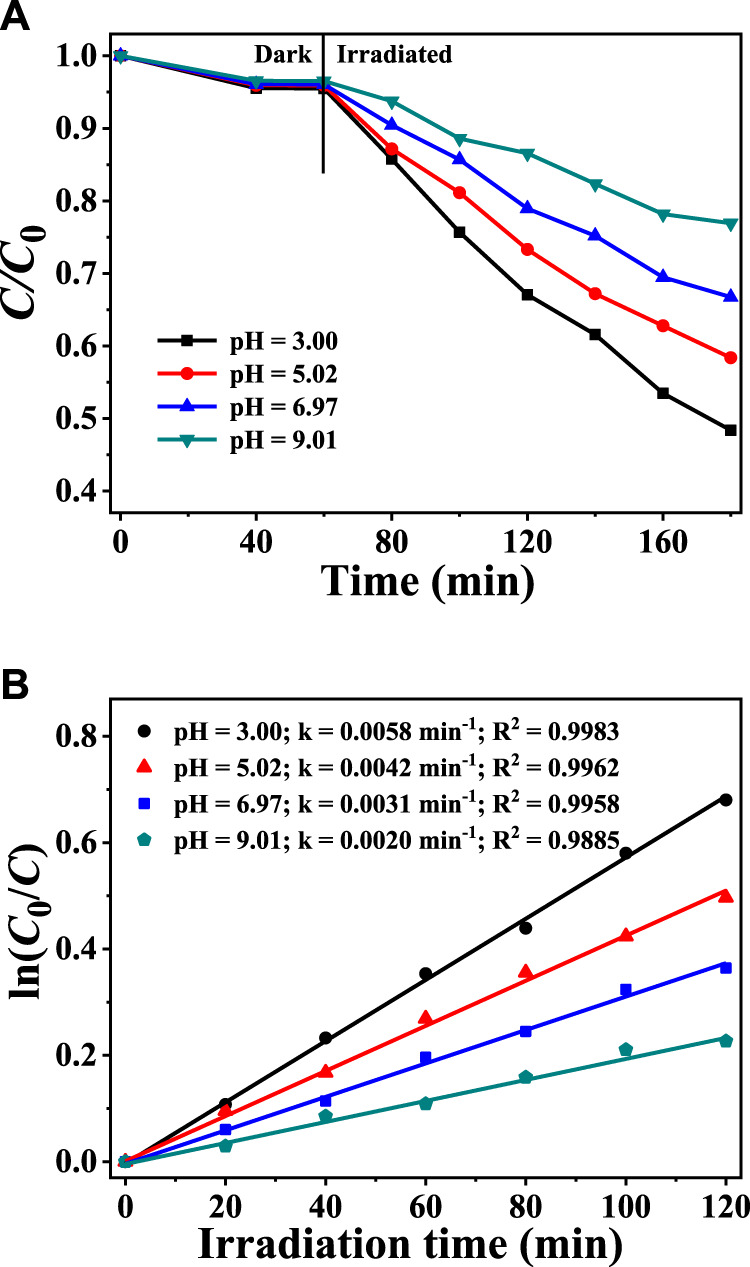
**(A)** Adsorption and photocatalytic degradation of CAP over CuInS_2_ in the mixed solution of CAP and Cr(VI) at different pH conditions **(B)** Corresponding kinetics plots for the photocatalytic CAP degradation reactions in **(A)**. ([Cr(VI)] = 10 mg/L, [CAP] = 10 mg/L, [catalyst] = 0.2 g/L).

As shown in Figure 6A, the pH value of the solution had little effect on the adsorption of CAP by CuInS_2_. However, the photodegradation rates of CAP can be remarkably affected by the pH of the solution. As shown in Figure 6B, when the solution’s starting pH is 3.0, the CAP degradation rate is the fastest at the given irradiation period, and the k value is 2.9 times that of pH 9.01. The rate of CAP degradation reduced as the pH of the solution increased, indicating that the more acidic solution promotes photocatalytic CAP degradation in the mixed solution system of CAP and Cr(VI). One reason for this is that the formed Cr(OH)_3_ tends to settle on the surface of the photocatalyst particles in neutral and alkaline solutions, reducing the available active sites of the photocatalysts and inhibiting further photocatalytic degradation of CAP and reduction of Cr(VI) (Wang et al., 2021a). The decrease in Cr(VI) conversion reduces the consumption of photogenerated electrons, causing more recombination of photogenerated electrons and holes and diminishing the photocatalytic oxidative degradation of CAP (Sun et al., 2021).

### Synergy of Photocatalytic Cr(VI) Reduction and CAP Degradation Over CuInS_2_

**FIGURE 7:**
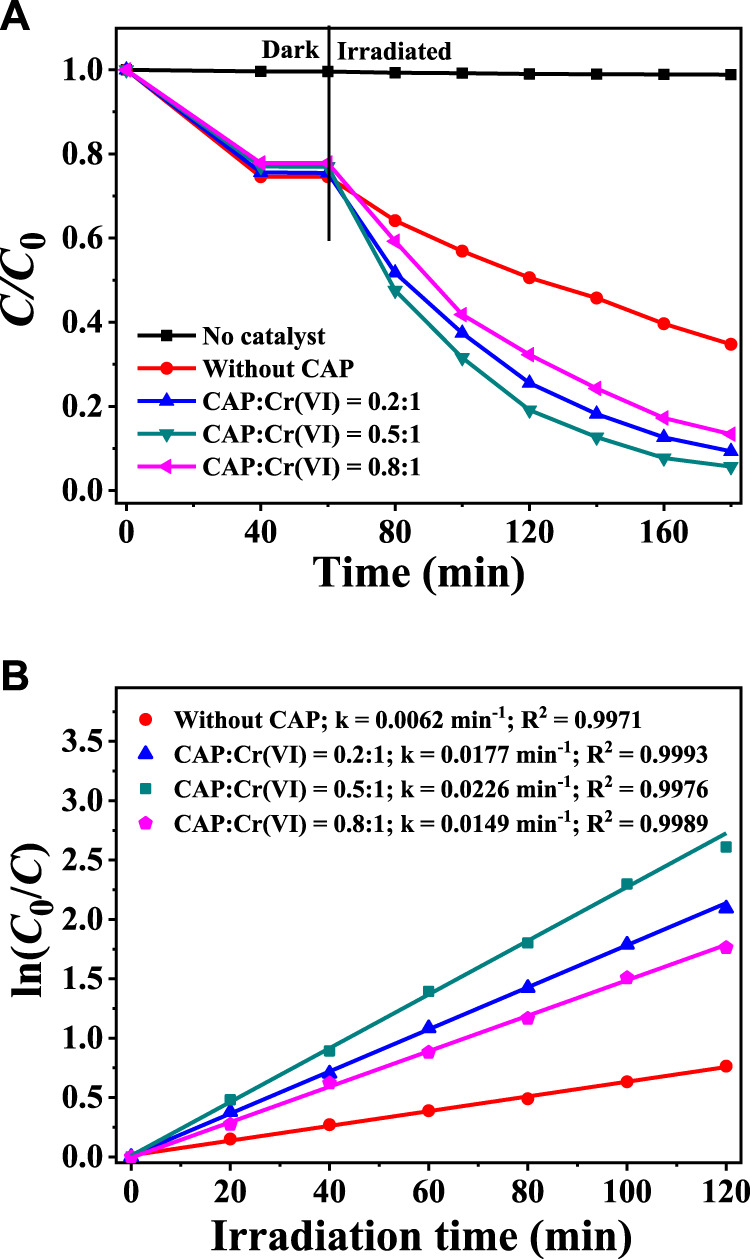
**(A)** Adsorption and photocatalytic reduction of Cr(VI) over CuInS_2_ under visible irradiation in the mixed solution with different weight ratios of Cr(VI) and CAP **(B)** Corresponding kinetics plots for the photocatalytic Cr(VI) reduction reactions in **(A)**. ([Cr(VI)] = 10 mg/L, [catalyst] = 0.2 g/L; pH = 3.0).

In the mixed solution of Cr(VI) and CAP, the possibility of simultaneous Cr(VI) reduction and CAP oxidation by the photocatalysis of CuInS_2_ was tested. The effects of different initial CAP concentrations on the photocatalytic reduction of 10 mg/L Cr(VI) over CuInS_2_ and the corresponding kinetic behaviors were studied. As illustrated in Figure 7A, without CuInS_2_, the reduction of Cr(VI) in the Cr(VI)/CAP mixed solution under visible-light irradiation can be neglected. In the mere Cr(VI) solution (without CAP), the photocatalytic reduction of Cr(VI) by CuInS_2_ under visible-light irradiation for 120 min removed only 77.5% of Cr(VI). When the CAP/Cr(VI) ratio was 0.5: 1, over 94.3% of Cr(VI) was decreased after 120 min of visible-light irradiation, implying that the presence of CAP might increase Cr(VI) reduction by serving as a photogenerated hole scavenger. Also, it can be observed that a variation in the CAP/Cr(VI) ratio can lead to a change in the Cr(VI) reduction rate. The optimal CAP/Cr(VI) ratio is 0.5: 1, and the *k* value is about 3.7 times that without CAP (Figure 7B). This might be attributed to that when CAP concentration rises, more CAP or intermediates would be adsorbed on the CuInS_2_ surface, potentially covering the catalyst’s active sites (Cherifi et al., 2021).

**FIGURE 8:**
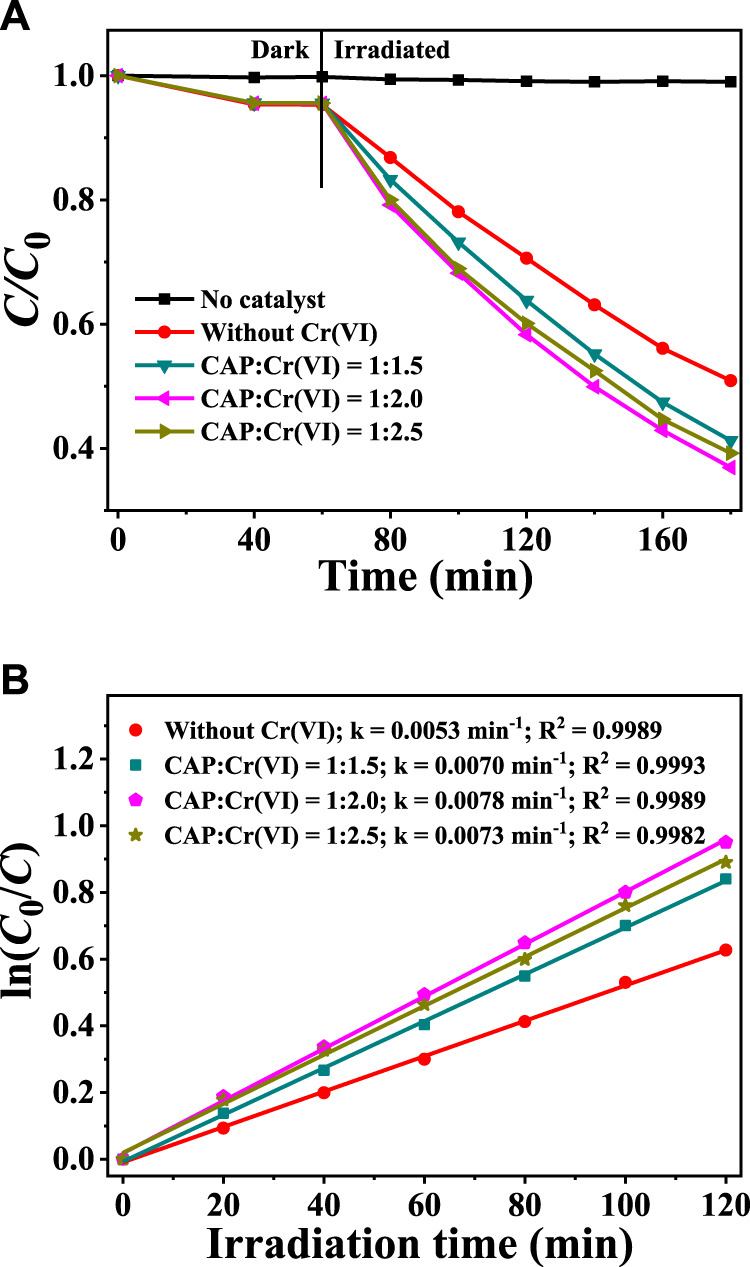
**(A)** Adsorption and photocatalytic degradation of CAP over CuInS_2_ under visible irradiation in the mixed solution with different weight ratios of Cr(VI) and CAP **(B)** Corresponding kinetics plots for the photocatalytic CAP degradation reactions in **(A)**. ([CAP] = 10 mg/L, [catalyst] = 0.2 g/L; pH = 3.0).

Besides, the effects of varying the initial Cr(VI) concentration on the degradation of 10 mg/L CAP over CuInS_2_ and the corresponding kinetic behaviors were examined. As shown in Figure 8A, CAP cannot be degraded without the presence of CuInS_2_ catalyst, and the coexistence of Cr(VI) can effectively improve the degradation rate of CAP in the presence of CuInS_2_ catalyst, similar to the effect of CAP on the reduction process of Cr(VI) in the mixed Cr(VI)/CAP solution under visible-light irradiation. The photodegradation kinetic behaviors of CAP over CuInS_2_ in the solutions containing different ratios of CAP and Cr(VI) were further explored, and the results are presented in Figure 8B. The k values increase with decreasing the CAP/Cr(VI) ratio from 1: 1 to 1: 2 and subsequently decrease with further decreasing the CAP/Cr(VI) ratio from 1: 2 to 1: 2.5. The optimized CAP/Cr(VI) ratio is 1: 2, with a k value (0.0078 min^−1^) for CAP degradation is about 1.5 times that of the mere CAP solution [without Cr(VI)]. The above results indicated that there is strong synergy between photocatalytic Cr(VI) reduction and CAP degradation over CuInS_2_.

### Possible Photocatalytic Mechanism

**FIGURE 9:**
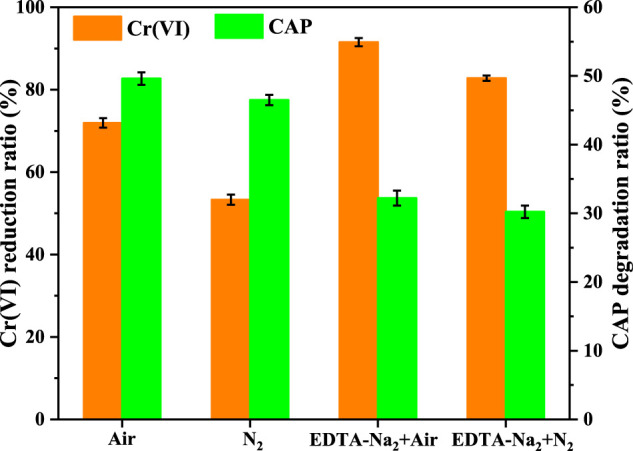
Cr(VI) reduction and CAP degradation over CuInS_2_ in the Cr(VI)/CAP mixed solution under air or N_2_ condition as well as with or without the addition of EDTA-Na_2_. ([Cr(VI)] = 10 mg/L, [CAP] = 10 mg/L, [catalyst] = 0.2 g/L, pH = 3.0, visible-light irradiation time = 120 min).

By carrying out the photocatalytic experiments with or without the addition of EDTA-Na_2_ (the scavenger for photogenerated holes) as well as in air or N_2_ environment, the mechanism of photocatalytic Cr(VI) conversion and CAP degradation over CuInS_2_ was investigated. Before the start of the experiments under the N_2_ environment, the reaction solution was purged with high-purity (> 99.999%) N_2_ for 1 h to eliminate the dissolved O_2_, and this process was maintained throughout the photocatalytic process. As shown in Figure 9, under visible-light illumination, the Cr(VI) conversion and CAP degradation over CuInS_2_ in the Cr(VI)/CAP mixed solution in the air environment were more efficient than those in the N_2_ environment. The suppressing impact of N_2_ environment was more noticeable in the case of Cr(VI) reduction, with the Cr(VI) reduction rate dropping from 71.9% in air to 53.3% in N_2_ environment. This is because that the interaction of dissolved O_2_ with photogenerated electrons can produce superoxide radicals (•O_2_) (Wang et al., 2016). •O_2_ can be further converted to H_2_O_2_ or disproportioned to •OH, which affects the Cr(VI) reduction and CAP degradation, respectively. Furthermore, it has been shown that •O_2_ is capable of reducing Cr(VI) to Cr(V), hence improving Cr(VI) conversion (Wei et al., 2016; Deng et al., 2017). As a consequence, the rates of Cr(VI) reduction and CAP oxidation in the air environment are higher than those in the N_2_ environment. As shown in Figure 9, the conversion proportion of Cr(VI) and the degradation proportion of CAP are 71.9% and 49.6%, respectively, without the addition of EDTA-Na_2_ in the air environment under visible-light irradiation for 120 min. After adding EDTA-Na_2_, the Cr(VI) conversion rose to 91.5% while the CAP degradation decreased to 32.2%. Because EDTA-Na_2_ can efficiently capture photogenerated holes, so enhancing photogenerated charge carrier separation and has a promoting influence on Cr(VI) reduction (Patnaik et al., 2018). On the other hand, the addition of EDTA-Na_2_ led to the decline in CAP degradation efficiency, suggesting that CAP degradation was mostly dependent on photogenerated holes (Qu et al., 2020). In the N_2_ environment, the improvement in the Cr(VI) conversion and the decrease in the CAP degradation were also observed after adding EDTA-Na_2_. Nevertheless, when the reaction was carried out in the N_2_ environment, EDTA-Na_2_ had a smaller promoting impact on the reduction of Cr(VI), owing to the reduced of O_2_/•O_2_^−^ mediated reduction in the decrease of dissolved O_2_ (Wang et al., 2016; Cherifi et al., 2021).

**FIGURE 10:**
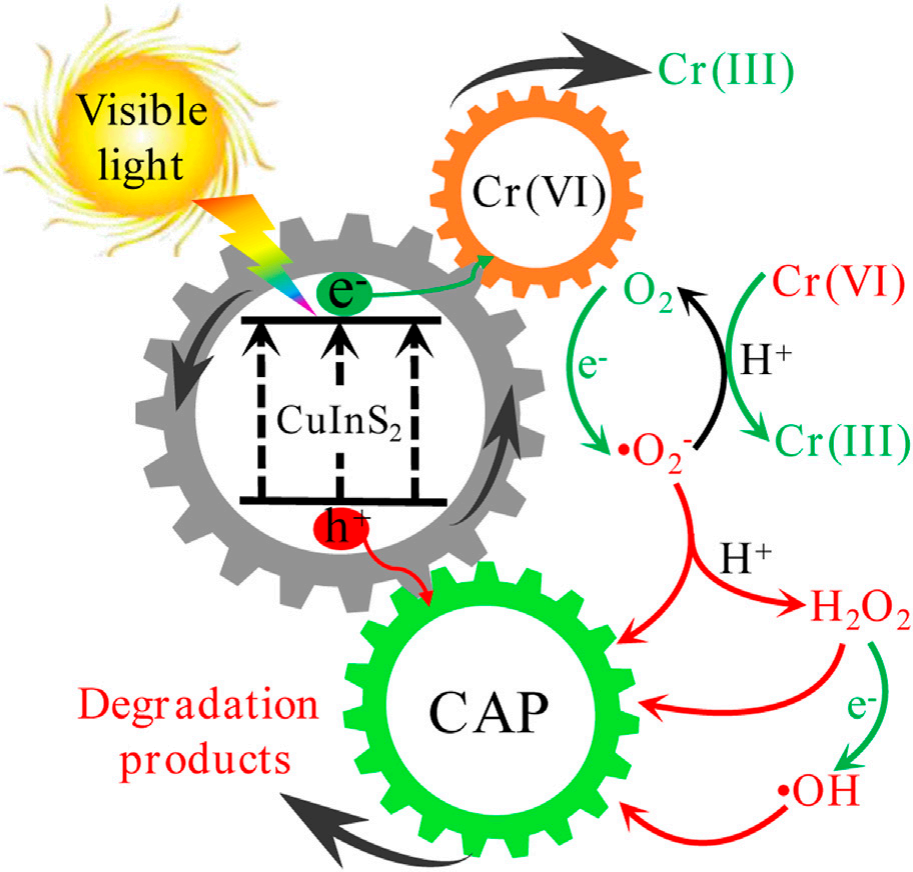
Possible mechanisms of concurrent photocatalytic Cr(VI) reduction and CAP degradation over CuInS_2_ under visible-light irradiation.

We postulated the possible mechanisms for the photocatalytic Cr(VI) reduction and CAP oxidation over CuInS_2_ as shown in Figure 10, based on the aforesaid results. Under visible-light irradiation, photogenerated electrons (*e* ^*−*^) and photogenerated holes (*h* ^*+*^) are produced respectively in the conduction band (CB) and valence band (VB) of CuInS_2_ (Eq. 3). Cr(VI) can be reduced by *e* ^*−*^ (Eq. 4), while CAP can be oxidized by *h* ^*+*^ (Eq. 5). The two simultaneous processes are capable of accelerating the separation of *e* ^*−*^ and *h* ^*+*^, resulting in a greater amount of *e* ^*−*^ for Cr(VI) reduction and *h* ^*+*^ for CAP oxidation (Liu et al., 2022c). In addition, *e* ^*−*^ can combine with dissolved O_2_ to form •O_2_^−^ (Eq. 6), and •O_2_^−^ can reduce Cr(VI) in the presence of H^+^ (Eq. 7) (Xia et al., 2018). Furthermore, •O_2_^−^ can combine with H^+^ to make H_2_O_2_ (Eq. 8), which subsequently reacts with *e* ^*−*^ to form the powerful oxidizing •OH (Eq. 9). Meantime, CAP may be oxidized by *h* ^*+*^ as well as the oxidizing species created, such as •O_2_^−^, •OH, and H_2_O_2_ (Eq. 10).

$$\mathrm{CuIn}S_{2}+hv\to \mathrm{CuIn}S_{2}\left(h_{vB}^{+}+e_{CB}^{-}\right)$$ (3)

$$\mathrm{Cr}\left(\mathrm{VI}\right)+3e_{CB}^{-}\to \mathrm{Cr}\left(\mathrm{III}\right)$$ (4)

$$h_{VB}^{+}+\mathrm{CAP}\to \mathrm{Degradation}\;\mathrm{Products}$$ (5)

$$O_{2}+e_{CB}^{-}\to •O_{2}^{-}$$ (6)

$$\mathrm{Cr}\left(\mathrm{VI}\right)+2•O_{2}^{-}+4H^{+}\to \mathrm{Cr}\left(\mathrm{III}\right)+2H_{2}\mathrm{O+}O_{2}$$ (7)

$$2•O_{2}^{-}+2H^{+}\to O_{2}+H_{2}O_{2}$$ (8)

$$H_{2}O_{2}+e_{CB}^{-}\to •\mathrm{OH+O}H^{-}$$ (9)

$$•O_{2}^{-}/H_{2}O_{2}/•\mathrm{ΟΗ+CAP}\to \mathrm{Degradation}\;\mathrm{products}$$ (10)

### Reusability and Stability of CuInS_2_ Photocatalyst

**FIGURE 11:**
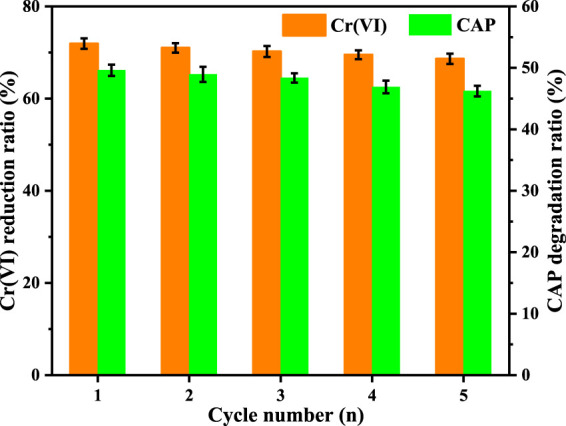
Cycle performance of our prepared CuInS_2_ in photocatalytic Cr(VI) reduction and CAP degradation in the Cr(VI)/CAP mixed solution. ([Cr(VI)] = 10 mg/L, [CAP] = 10 mg/L, [catalyst] = 0.2 g/L, pH = 3.0).

**FIGURE 12:**
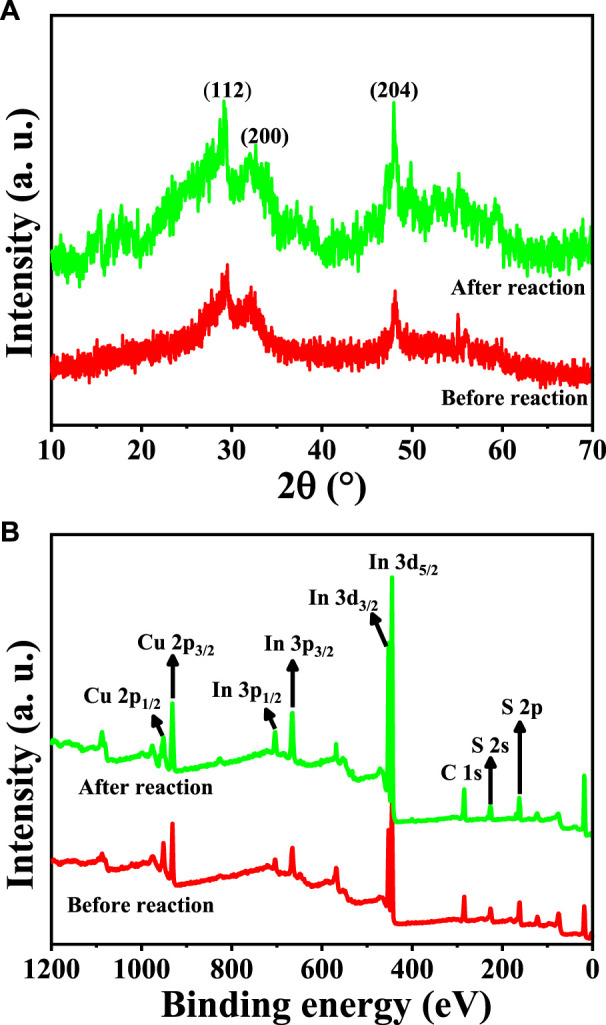
**(A)** XRD patterns and **(B)** survey XPS spectra of the CuInS_2_ before and after the cycle tests.

The photocatalytic activity and durability of a catalyst are equally significant in practical applications. The photocatalytic endurance of CuInS_2_ was tested by performing five successive cycles of Cr(VI) reduction and CAP degradation in the mixed Cr(VI)/CAP solution by the same process as mentioned above, but 40 mg of photocatalyst and 200 ml of mixture were used. When each cycle ended, the photocatalyst was collected, washed and dried at 80°C for 12 h. In each cycle test, a certain amount of original Cr(VI)/CAP mixture was injected to maintain the initial concentration of pollutants. As indicated by Figure 11, both the Cr(VI) reduction rate and the CAP degradation rate decrease only a bit as the cycle number rises. The reduced percentage of Cr (VI) and the degraded percentage of CAP are in turn 71.9% and 49.6% in the first cycle, but still 68.7% and 46.2% in the fifth cycle, respectively. Thus, the CuInS_2_ photocatalyst has been shown to have fair reusability for synchronous photocatalytic Cr(VI) conversion and CAP degradation. Figures 12A,B show the XRD patterns and survey XPS spectra of the CuInS_2_ before and after the reuse tests. As can be seen from Figures 12A,B, the peak number and location of the CuInS_2_ after the reuse tests are virtually identical to those of fresh CuInS_2_, showing that the crystal structure, composition and elemental valence of CuInS_2_ have little change. Accordingly, CuInS_2_ appears to have strong stability and fair reusability, which bodes well for its future use in wastewater treatment.

## Conclusion

CuInS_2_ nanoparticles were synthesized by a straightforward solvothermal method and explored as a photocatalyst in the simultaneous photocatalytic Cr(VI) reduction and CAP oxidation under visible-light irradiation. It was demonstrated that lower pH and oxygenated atmosphere are advantageous for Cr(VI) reduction and CAP oxidation. The simultaneous photocatalytic reduction of Cr(VI) and oxidation of CAP over CuInS_2_ in the mixed Cr(VI)/CAP solution had synergistic effect, which was more efficient than only the photocatalytic reduction of Cr(VI) and only the photocatalytic oxidation of CAP. Furthermore, after five runs, the CuInS_2_ sample retains a high rate of mixed pollutant removal. The possible mechanisms for the simultaneous photocatalytic reduction of Cr(VI) and oxidation of CAP over CuInS_2_ were proposed. The results of this work may shed light on the synergistic effect of Cr(VI) reduction and CAP oxidation on the CuInS_2_ catalyst. This study shows that CuInS_2_ is a potential high-performance visible-light photocatalyst for treatment of organic contaminants and heavy metal ions in water at once.

## Data Availability

The original contributions presented in the study are included in the article/Supplementary Material, further inquiries can be directed to the corresponding authors.
